# Possible Coexistence of Pellagra in a Malnourished Patient with Seizure and Multiple Cerebrovascular Foci: A Case Report

**DOI:** 10.3390/reports8020062

**Published:** 2025-05-04

**Authors:** Hanako Aoki, Toshiki Uchihara, Yoshinori Ito

**Affiliations:** 1 Department of Neurology, Yokufukai Hospital, 1-12-1 Takaidonishi, Suginami-ku, Tokyo 168-8535, Japan; neurologicien@yahoo.co.jp (T.U.); itoh-y@yokufu-hp.jp (Y.I.); 2 Department of Neurology and Neurological Science, Institute of Science Tokyo, 1-5-45 Yushima, Bunkyo-ku, Tokyo 113-8519, Japan; 3 Department of General Internal Medicine, Okinawa Chubu Hospital, 281 Miyasato, Uruma, Okinawa 904-2293, Japan

**Keywords:** pellagra, niacin, seizure, malnourished patient, multiple cerebrovascular foci

## Abstract

Background and Clinical Significance: Pellagra is caused by a chronic deficiency of niacin (vitamin B3 or nicotinic acid): it is rare in developed countries, where the major risk factors are chronic alcoholism and intestinal malabsorption. Although it typically presents three main symptoms, dermatitis, diarrhea, and dementia, some cases do not show these classic symptoms. Case Presentation: We report a case of a malnourished patient with seizure and multiple cerebrovascular foci, in whom a postmortem autopsy revealed the findings of pellagra. The patient had atypical symptoms of seizure as pellagra and the multiple cerebrovascular lesions, which made the diagnosis difficult. Conclusions: The aim of this paper is to recognize the importance of suspecting pellagra as a treatable disease, especially when patients with eating disorder present atypical symptoms.

## 1. Introduction and Clinical Significance

Pellagra is a nutritional disorder caused by niacin (vitamin B3) deficiency, classically characterized by the “four Ds”: diarrhea, dermatitis, dementia, and death [1]. The disease primarily affects malnourished populations, alcoholics, and psychiatric patients [2]. While pellagra was once endemic in the United States, it has become rare in developed countries due to improved nutrition and food fortification. However, recent reports suggest a potential resurgence linked to HIV infections and dietary trends, including eating disorders or self-imposed dietary limitations [3,4,5,6].

The clinical presentation of pellagra includes a characteristic photodistributed rash, gastrointestinal symptoms, and neuropsychiatric disturbances [1]. Among the central nervous system (CNS) manifestations, cognitive impairment is the most widely reported, with various forms of dementia documented [7,8]. These cognitive deficits are often accompanied by hallucinations, anxiety, depression, seizures, and parkinsonian motor symptoms [9,10]. Previous case reports have highlighted diverse neurological presentations, including a patient who initially exhibited seizures before being diagnosed with pellagra based on the characteristic triad of symptoms [11]. Another case presented with progressive dermatitis and dysphagia but lacked both gastrointestinal and neuropsychiatric symptoms [12]. The early diagnosis and treatment of pellagra are critical, as the disease can be fatal if left untreated. However, diagnosis remains challenging because the classical triad of symptoms is not always present. Confirmation typically involves clinical improvement following niacin administration, supported by a urinary metabolite analysis [13].

We present a case of pellagra which presented with seizures and was suspected to be pellagra on the postmortem neuropathology. This case could not be diagnosed because of a concomitant cerebrovascular disorder. This patient experienced a decline in mood, leading to a reduced food intake and worsening nutritional status. Neurologic symptoms were common symptoms of pellagra, but no other symptoms of dermatitis or gastrointestinal disorders were present. This case report highlights an unusual neurological presentation of pellagra, underscoring the importance of considering niacin deficiency in patients with unexplained neuropsychiatric and systemic symptoms.

## 2. Case Report

### 2.1. Clinical Presentations

**Figure 1:**
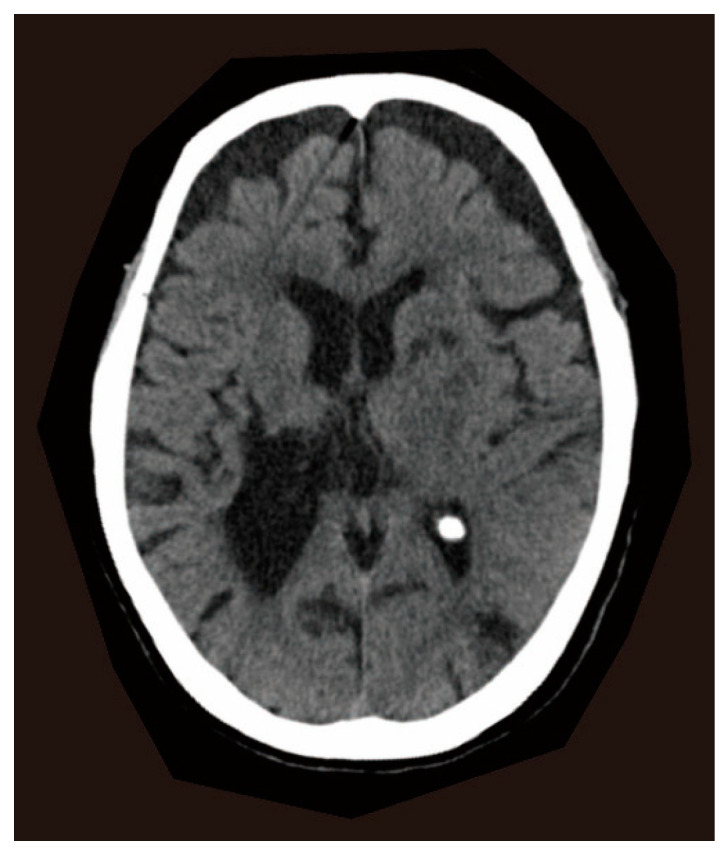
Computed tomography (CT) brain imaging. The CT image shows hypodensities in the right striatum, bilateral caudate nucleus, left anterior internal capsule, and left occipital lobe, suggesting old hemorrhage and ischemic foci.

A 77-year-old woman presented to the emergency department with her first seizure, a generalized seizure with a 30 s tonic–clonic component. When she arrived at our hospital, her seizure had stopped, and her level of consciousness was clear. Computed tomography (CT) of the brain showed an old hemorrhage in the right striatum and old ischemic foci in the bilateral caudate nuclei, left anterior internal capsule, and left occipital lobe (Figure 1). General blood tests were normal. A possible late seizure associated with old cerebrovascular diseases was suspected, and an antiepileptic medication, levetiracetam, was initialized. She was discharged after stabilization without further seizures. Five months later, the antiepileptic drug was discontinued due to an impaired consciousness. However, 3 days later, a second seizure occurred and levetiracetam was restarted. At this time, no new abnormalities were detected in the general blood tests and brain CT. Her medical history included a right striatal hemorrhage at age 49, which caused hemiplegia and was surgically removed. She had no history of alcoholism or seizure. During the past 5 years, she had been depressed, decreased her food intake, and lost 20 kg. She had been treated with nitrazepam and mirtazapine for depression until two years before admission. However, at the time of hospitalization, she was not taking any medications associated with neuropathological changes. Her physical examination revealed a thin body, suggesting a malnourished state. She had no diarrhea or dermatitis. During these hospitalizations, no specific nutritional evaluations, such as serum vitamin or niacin levels, or urinary amino acid testing, were performed, as pellagra was not clinically suspected. After the second seizure, the seizures did not recur, but she continued to be malnourished and died of pneumonia at age 78.

### 2.2. Autopsy Findings

**Figure 2:**
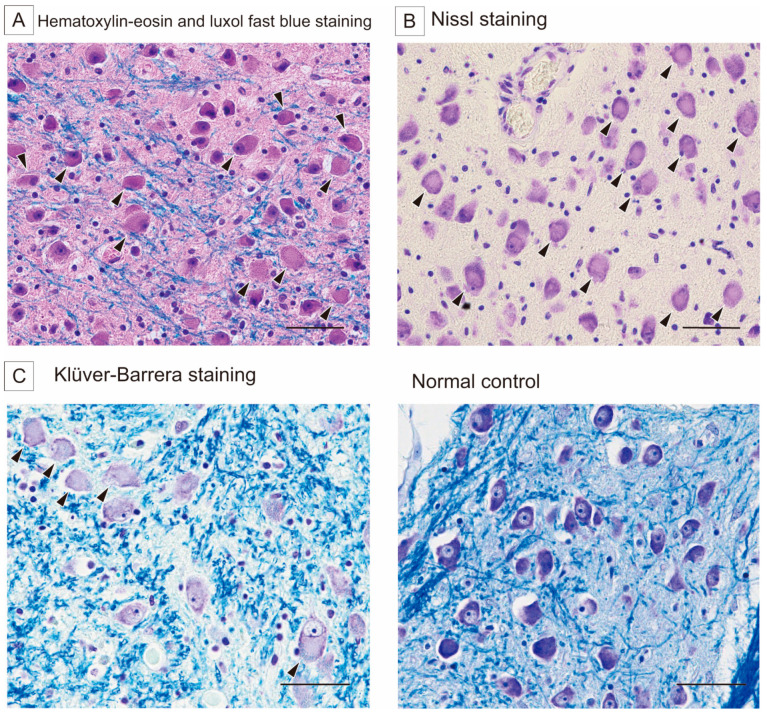
Histological images of the pontine base. (**A**) Hematoxylin–eosin and luxol fast blue staining shows numerous swollen neurons (arrowheads). Scale bar indicates 100 μm. (**B**) Nissl staining shows central chromatolysis (arrowheads). Scale bar indicates 100 μm. (**C**) High magnification of Klüver–Barrera staining reveals the central chromatolysis (arrowheads, **left**) in comparison with a normal control (**right**). Scale bar indicates 100 μm.

At autopsy, the microscopic findings included numerous swollen neurons in the pontine base without neuronal loss or gliosis (Figure 2). Slightly swollen neurons were found in the cyngulate gyrus, motor cortex, amygdala, and insular gyrus. The swollen neurons were the most conspicuous in the pontine nucleus, showing central chromatolysis by Nissl and Klüver–Barrera staining (Figure 2) and negativity for Gallyas–Braak silver staining, alpha-synuclein, and SMI-31. These neuropathological findings were compatible with the cytopathological changes characteristic of pellagra. In retrospect, the seizures and longstanding depressed state might have been related to pellagra as well as the multiple cerebral vascular lesions.

In addition to the brain, other tissues typically affected in pellagra, such as the skin and gastrointestinal tract, were examined by certified pathologists, but no gross or histological findings suggestive of pellagra were identified in these organs.

## 3. Discussion

The typical clinical symptoms of pellagra are characterized by dermatitis, dementia, and diarrhea [1]. The rapid diagnosis of pellagra is based on the combination of typical clinical symptoms and low niacin levels. However, the severity of each symptom varies from patient to patient, and some may not appear at all. Pellagra can also present with neurological abnormalities such as encephalopathy, seizure, peripheral neuropathy, and mental disorders [14]. When patients present with atypical and nonspecific symptoms, the rarity of pellagra makes it difficult to include in the differential diagnosis. Therefore, some cases are diagnosed only at autopsy [15]. In recent years, there has been an increasing number of case reports supporting the re-emergence of diseases associated with nutritional deficiencies, such as pellagra [16]. This case highlights the importance of considering nutritional-deficiency-related diseases, such as pellagra, as a differential diagnosis when encountering some neurological symptoms in malnourished patients, especially patients with underlying risk factors such as eating disorders, alcohol dependence, and dietary restrictions. As a treatable condition, the accurate evaluation and diagnosis of pellagra are essential for preventing irreversible complications and mortality.

The neuropathological characteristic of pellagra is the central chromatolysis of neurons in various areas, but especially in the pontine nuclei and Betz cells [15]. The pontine nuclei were heavily involved in alcoholic pellagra, and only the pontine nuclei were involved in some patients [17]. In the experimental mouse models using 6-aminonicotinamide, an antagonist of niacin, anterior horn cells in the spinal cord and motor neurons in the brain showed the ultrastructural features of neuronal chromatolysis [18]. These findings suggest that niacin deficiency can lead to the characteristic neuronal changes observed in human pellagra. Interestingly, it has been reported that prolonged sleep deprivation can also induce the chromatolysis of central nervous system neurons, and this neuronal damage is notably similar to that seen in pellagra. It has been hypothesized that sleep deprivation may lead to nicotinic acid depletion, resulting in secondary nicotinamide adenine dinucleotide (NADH) and adenosine triphosphate exhaustion, which may underlie the observed neuronal changes. Niacin, a collective term that includes both nicotinic acid and nicotinamide, plays a crucial role in cellular energy metabolism as a precursor of NADH. Therefore, the neuronal changes associated with prolonged sleep deprivation reinforces the notion that neuronal alterations in pellagra could result from niacin deficiency [19].

In this patient, the presence of numerous swollen neurons in the pontine nuclei and cerebral cortex was compatible with pellagra, although the clinical manifestations were not specific enough to suggest pellagra. Concurrent multiple cerebrovascular lesions might have obscured clinical suspicion of this rare but treatable condition. Pellagra should be considered as a possible therapeutic intervention not only in alcoholics but also in patients with a prolonged dietary deficiency who present with even nonspecific neurological symptoms. Although the observed neuropathological findings were characteristic of pellagra, it should be noted that nutritional deficiencies often coexist, and a comprehensive evaluation is essential when considering overlapping features with other disorders such as Wernicke encephalopathy.

While the diagnosis of pellagra in this case was made postmortem, the morphological findings are unlikely to be explained solely by postmortem changes. A recent systematic review on postmortem changes in the brain cell structure reported that early postmortem changes include fluid shifts, cellular vacuolization, and membrane deformation, while later changes involve the progressive loss of membrane integrity and the eventual loss of visualization of the cells [20]. However, chromatolysis has not been described as a common postmortem phenomenon. In addition, generalized signs of postmortem degeneration were not prominent in our case. Therefore, the presence of selective chromatolysis in the absence of typical postmortem degradation supports the interpretation that this is a pathological feature of pellagra rather than a postmortem artifact.

## 4. Conclusions

This case highlights the importance of considering pellagra, even in the absence of typical symptoms, especially in malnourished patients or those with underlying conditions like eating disorders. While the presentation with seizures and cerebrovascular lesions underscores the complexity of the diagnosis of pellagra, recognizing the potential for reversible nutritional deficiencies is crucial. Because pellagra is a treatable disease, early diagnosis and prompt treatment can significantly affect patient well-being and prognosis. The re-emergence of diseases related to nutrition disorders, including pellagra, necessitates heightened awareness.

## Data Availability

The data presented in this study are available upon request from the corresponding author. The data are not publicly available due to privacy concerns.
